# Posterior Dynamic Stabilization with Limited Rediscectomy for Recurrent Lumbar Disc Herniation

**DOI:** 10.1155/2021/1288246

**Published:** 2021-12-23

**Authors:** Lei Luo, Chen Zhao, Pei Li, Liehua Liu, Qiang Zhou, Fei Luo, Lichuan Liang

**Affiliations:** ^1^Department of Orthopedics, The Third Affiliated Hospital of Chongqing Medical University, Chongqing 401120, China; ^2^Department of Orthopedics, Southwest Hospital, Army Medical University, Chongqing 400038, China

## Abstract

**Objective:**

Recurrent lumbar disc herniation (RLDH) is the most common cause of sciatica after primary discectomy. The purpose of this study was to evaluate the efficacy of transpedicular dynamic stabilization (TDS) combined with limited rediscectomy in the treatment of single-level RLDH.

**Methods:**

We retrospectively evaluated a consecutive series of 24 middle-aged patients who underwent TDS (Dynesys system) combined with limited rediscectomy (i.e., removing only extruded or loose disc fragments) for single-level Carragee type II and type IV RLDH between April 2012 and September 2017. Clinical results were evaluated with visual analog scale (VAS) for leg and low back pain, Oswestry Disability Index (ODI) scores, and complications. Imaging data include lumbar segment motion and intervertebral height.

**Results:**

The mean follow-up period was 38 months. The VAS and ODI scores were significantly improved at the last follow-up. The average range of motion (ROM) at the stabilized segment was 6.4° before surgery and 4.2° at the last follow-up, with a 78.6% mean preservation (*P* < 0.05). Intervertebral height at the stabilized segment decreased slightly after surgery (*P* < 0.05). However, there was no further decline at the last follow-up. There were no cases of reherniation, screw loosening, or segmental instability.

**Conclusions:**

TDS combined with limited rediscectomy resulted in an effective procedure in middle-aged patients with Carragee type II and type IV RLDH. It was able to stabilize the operated segment with partial motion preservation. Moreover, it could maintain disc height and decrease the risk of recurrence in patients with a large posterior annular defect.

## 1. Introduction

Discectomy is considered as the main surgical method in patients with lumbar disc herniation (LDH) without segmental instability [1]. Nevertheless, recurrent lumbar disc herniation (RLDH) is one of the most common complications that can cause severe pain and reoperation after primary discectomy, with reported incidence ranging from 7% to 24% [2]. The definition of RLDH has varied among different authors. In most studies, recurrent lumbar disc herniation was defined as a disc hernia at the same level of a previous discectomy in patients with a pain-free interval of at least 6 months long after surgery, regardless of ipsilateral or contralateral herniation [3]. Numerous factors have been associated with an increased rate of reherniation following primary discectomy. Lumbar instability and increased stress upon the intervertebral disc after discectomy may be the major controllable risk factors for RLDH [4].

Surgical treatment for RLDH is indicated in patients with continuous and severe pain, resistant to conservative treatment or cases with motor deficiency. Traditional surgical options include revision discectomy or discectomy supplemented with instrumented fusion. According to the reports by Buchmann et al. [5] and Hou et al. [6], rediscectomy provided good to excellent pain relief in 55%–96% of the patients. Because of peridural adhesion, extension interlaminar fenestration would be necessary to reduce the risk of dural and nerve root injury. However, excessive laminectomy may result in lumbar instability. In addition, the intervertebral height decreases after rediscectomy, which can lead to the progression of disc degeneration [7]. Therefore, some surgeons advocate the routine use of instrumented fusion in the treatment of RLDH [8]. Intervertebral fusion can maintain lumbar stability and restore intervertebral height. A recent review by Dower et al. [9] demonstrated that fusion resulted in a statistically significant improvement in back pain compared with discectomy (60.1% vs. 47.2%, respectively). Nonetheless, intervertebral fusion sacrifices the activity of the fixed segment and may accelerate degeneration of adjacent segments [10].

Based on the above deficiencies, transpedicular dynamic stabilization (TDS) was introduced as an alternative to fusion to preserve the activity of the instrumented segments while stabilizing the lumbar spine. Moreover, this technique is targeted to maintain the intervertebral height and reduce the mechanical load upon the disc. It is reported that dynamic stabilization is useful to prevent or diminish recurrent disc herniation after discectomy [11]. Therefore, we proposed TDS combined with limited rediscectomy (i.e., removing only extruded or loose disc fragments) [12] for the treatment of patients with RLDH as an alternative to instrumented fusion. The purpose of this study is to assess the clinical outcomes of TDS with the Dynesys system (Zimmer, USA) for RLDH.

## 2. Materials and Methods

### 2.1. Patients

This retrospective study included 24 consecutive patients who underwent Dynesys stabilization combined with limited rediscectomy for single-level RLDH from April 2012 to September 2017. There were 16 men and 8 women with an average age of 47.6 years (range of 32–62 years). This study has been approved by the Ethical Committee of the Third Affiliated Hospital of Chongqing Medical University (SKYW20190316). The study was conducted per the ethical principles that have their origins in the Declaration of Helsinki and its subsequent amendments. Informed consent was obtained from all patients. Inclusion criteria were as follows: (1) age ≥30 years at the time of surgery; (2) patients with symptoms of new onset low back pain and radicular leg pain and/or decreased muscular strength and/or abnormal sensation; (3) diagnosis of single-level RLDH confirmed by MRI (primary surgery included open discectomy or microendoscopic discectomy); (4) Carragee type II (presence of extruded or sequestered fragments with wide annular rupture; rupture ＞6 mm) or type IV herniation (the width of the basilar part of the herniated disc >6 mm; annulus is intact and without free fragments under the annulus) [13]; (5) failed in at least 6 weeks of conservative treatment (oral medication, physical therapy, and so on); and (6) underwent the operation of TDS (Dynesys, Zimmer Spine) combined with limited rediscectomy. Exclusion criteria included the following: (1) more than 1/2 reduction in intervertebral height; (2) rigid segmental kyphosis; and (3) osteoporosis (T-score ≤−2.5, DEXA).

### 2.2. Surgical Procedure

**Figure 1:**
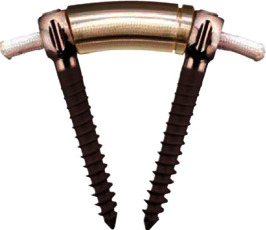
The Dynesys system consists of titanium alloy screws, PET cords, and PCU spacers.

The Dynesys system is a pedicle screw-based dynamic stabilization system. In the system, titanium alloy pedicle screws are connected by a polyethylene terephthalate (PET) cord that goes through the center of a hollow cylinder polycarbonate urethane (PCU) spacer instead of the traditional rigid rod (Figure 1). By appropriately tightening the cord and selecting the length of the spacer, dynamic stabilization would be achieved in the instrumented segment [14].

Patients were placed in the prone position under general anesthesia. A midline dorsal incision in the skin, subcutaneous tissue, and lumbodorsal fascia was applied. Extended interlaminar fenestration decompression was performed through the posterior median approach on the symptomatic side. Only extruded or loose disc fragments were removed. Pedicle screws were inserted through the Wiltse approach under imaging control. The entry point was located at the junction of the lateral border of the superior articular process and the basilar part of the transverse process. Then, the patients' position was modified to obtain the appropriate lumbar lordosis. The spacer was cut according to the measured distance between the screws (distraction force of 1.5 N). The central cord and the spacer were then locked within the screw heads. Patients received a soft support lumbar corset for 3 weeks after surgery.

### 2.3. Clinical and Radiologic Evaluation

Clinical outcomes were assessed with visual analog scale (VAS) for low back and leg pain and Oswestry Disability Index (ODI). Operative time, blood loss, and complications were also documented. Standing plain and dynamic radiographs with flexion and extension views were obtained preoperatively, at 3 months postoperatively, and the last follow-up. The evaluation index included the lordosis at the instrumented segment, the height of the intervertebral disc, and range of motion (ROM) at the instrumented level, the 1^st^ proximal segment, and the lumbar spine (L1-S1). Disc height (DH) was calculated using the anterior intervertebral space height (AH) and posterior intervertebral space height (PH) on the lateral standing lumbar X-ray: (AH + PH)/2. Segmental ROM was calculated as the angle difference value between the inferior surface of the upper vertebrae and the superior surface of the lower vertebrae on the lateral standing lumbar flexion-extension X-ray. Disc degeneration grade was evaluated according to the Pfirrmann classification on T2-weighted sagittal and axial MRI [15].

### 2.4. Statistical Analysis

Statistical analysis was conducted using SPSS (version 16.0, SPSS Inc.). The clinical and radiologic results were analyzed using two-way ANOVA. Significance was defined as *P* < 0.05.

## 3. Results

### 3.1. Perioperative Data and Complications

The mean interval between the primary and revision surgeries was 66.0 ± 53.2 months (range of 6–192 months). The RLDH level was L4/5 in 14 (58.3%) and L5/S1 in 10 (41.7%) patients (Table 1). The mean operative time was 136 minutes (range of 98–183 minutes), with an average blood loss of 266 ml (range of 100–500 ml). The mean follow-up duration was 38 months (range of 28–63 months). Superficial incision infection was observed in one patient 6 days after surgery. The patient was cured by debridement and antibiotics. One patient developed low back and hip pain 3 weeks after surgery, which was relieved after 10 days of conservative treatment. There were no cases of reherniation, screw loosening, or dural and nerve root injury.

### 3.2. Clinical Outcome

The mean VAS scores for low back pain decreased from 3.8 ± 0.8 (range of 3–5) preoperatively to 1.3 ± 0.6 (range of 0–2) at 3 months postoperatively and 0.9 ± 0.4 (range of 0–2) at the last follow-up. The VAS scores for low back pain were significantly improved at the final follow-up evaluation compared with the baseline values (*P* < 0.05). Similar to the VAS scores for low back pain, the mean VAS scores for leg pain decreased from 5.5 ± 1.1 (range of 4–8) to 0.9 ± 0.6 (range of 0–2) at 3 months postoperatively and 0.7 ± 0.5 (range of 0-1, *P* < 0.05) at the last follow-up. The mean ODI was 57.9% ± 10.6% (range of 40%–76%) preoperatively, 23.2% ± 7.8% (range of 6%–40%) at the 3-month follow-up, and 12.8% ± 6.2% (range of 0%–24%) at the last follow-up. The changes in VAS_leg_ and ODI scores between the preoperative period and the follow-ups were statistically significant as well (*P* < 0.05) (Table 2).

### 3.3. Radiologic Outcome

**Figure 2:**
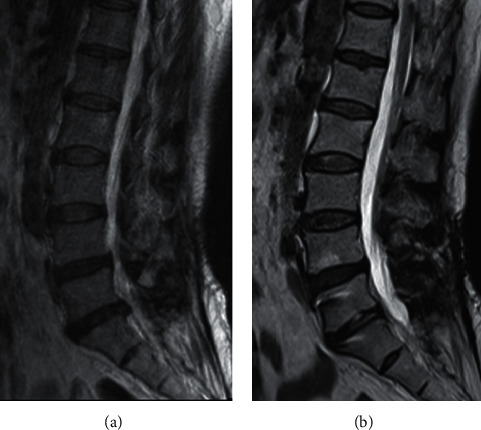
A case of a 53 years old female patient with RLDH at L5-S1. MRI scans showed rehydration at 22-month follow-up after transpedicular dynamic stabilization.

The lordosis at instrumented segment was reduced from 8.0° (range of −5.7° to −13.3°) before surgery to 6.8° (range of 2.3°–10.8°) at the 3 months follow-up (*P* ＜ 0.05) and 7.0° (range of 2.6°–11.3°) at last follow-up (*P* ＞ 0.05). The average disc height decreased slightly from preoperative 10.4 mm (range of 6.8 mm–13.8 mm) to 9.3 mm (range of 6.3 mm–12.6 mm) at 3 months postoperatively (*P* < 0.05). There was no further decline at the last follow-up (*P* > 0.05). The average ROM at instrumented segment was 6.4° (range of 3.1°–17.3°) before surgery, 4.0°(range of 2.6°–5.9°) at 3 months after surgery, and 4.2° (range of 3.0°–5.2°) at last follow-up. Compared with preoperatively, 78.6% (range of 24%–152%) of ROM was preserved at the last follow-up. The ROM at the 1^st^ proximal segment was 9.0° (range of 3.5°–16.0°) before surgery, 9.5° (range of 5.8°–15.7°) at 3 months after surgery, and 9.9° (range of 5.5°–14.9°) at the last follow-up. The differences were not statistically significant (*P* > 0.05). The lumbar motion was reduced from 34.1° (range of 13.2°–60.4°) before surgery to 28.8° (range of 17.2°–40.4°) at 3 months after surgery (*P* < 0.05) and 34.8° (range of 18.7°–63.2°) at last follow-up (*P* > 0.05) (Table 3). MRI was performed in 11 patients during the follow-up period. Of the 11 patients, the disc degeneration grade (Pfirrmann classification) improved at the index level was observed in 6 patients (Figure 2). The other 5 patients demonstrated no visible signal intensity change at the index level. No progressive degeneration was noted at the first proximal segment in the 11 patients.

## 4. Discussion

The optimal treatment for RLDH remains controversial. Several studies demonstrated that rediscectomy could be able to achieve satisfactory clinical results [3, 6, 16]. However, the chance of segmental lumbar instability increases as rediscectomy often requires more aggressive laminectomy and facetectomy for better exposure of the nerve root canal [17, 18]. Moreover, excessive sagittal activity at the involved segment after discectomy is a risk factor for recurrent lumbar disc herniation [19, 20]. Furthermore, large posterior annular defect is prone to develop recurrent disc herniation. According to the study by Carragee et al., the recurrence rate after discectomy in patients with Carragee type II and IV herniations was 27% and 38%, respectively [13]. Discectomy alone might be insufficient to achieve satisfactory results. Thus, we performed posterior dynamic stabilization with limited rediscectomy for the treatment of Carragee type II and type IV RLDH to stabilize the lumbar spine, reduce excessive intervertebral motion, and decrease the risk of re-recurrent disc herniation.

The Dynesys system is supposed to stabilize the operated segment with partial motion preservation. The biomechanical analysis demonstrated that the Dynesys system reduced the intersegmental motion in flexion, extension, lateral bending, and axial rotation compared with structurally damaged specimens so that it could provide substantial stability in case of lumbar degenerative disease [21, 22]. We investigated the clinical and radiologic results of patients undergoing TDS and limited rediscectomy for RLDH. In general, patients had clinically and statistically significant improvements in VAS_back,leg_ and ODI scores. In addition, flexion/extension radiographs showed significant preservation of ROM at the stabilized segment without lumbar instability or spondylolisthesis. Some studies have also shown that the Dynesys system provides the lumbar spine with sufficient stability in treating degenerative spondylolisthesis [23, 24]. In the long term, there is always a concern of screw loosening in patients treated with dynamic stabilization [25] although the loosened screws appeared to be asymptomatic [26]. In the present study, no cases of screw loosening were found at the last follow-up. The reasons might be the patients with osteoporosis were excluded, and minimal bone resection as well as pedicle screws placement lateral to the facet joints that makes the rotation axis of the Dynesys system close to the rotation axis of the motion segment could reduce the stress on the system.

Disc removal may lead to accelerated disc degeneration at the operative level. Disc height reduction and endplate degeneration may be the most common findings following discectomy [27, 28]. Excessive removal is associated with the progression of disc space narrowing, which may lead to low back pain over time [29]. Conversely, limited nucleus pulposus removal, preserved disc height, and moderate disk degeneration are significant risk factors for RLDH [19, 30, 31]. Therefore, surgical treatment which can both preserve the disc height and decrease the incidence of reherniation may allow for improved outcomes. According to the literature, discectomy with additional transpedicular dynamic stabilization is useful to prevent progression of intervertebral disc degeneration and decrease the risk of recurrence in treating primary lumbar disc herniation [32, 33]. Our results showed that disc height decreased slightly after TDS and limited discectomy, but there was no further decline at the last follow-up. The disc height could be maintained at the last follow-up compared with the postoperative value. The result may indicate that dynamic stabilization can delay or prevent the progression of disc degeneration. Of the 11 patients who underwent MRI examination during the follow-up period, 6 patients demonstrated improved disc degeneration grade at the index level, whereas the other patients showed no visible signal intensity change at the index level. In addition, a second recurrence did not occur in any patients in this study at the last follow-up. This further confirms that the Dynesys system can decelerate the degeneration process. However, its mechanism remains unclear. One possible mechanism is that intradiscal pressure is reduced by axial distraction [34], and moderate dynamic compression or distraction could promote anabolism in nucleus pulposus cells [35, 36], thus allowing the disc to repair itself. Some studies reported disc rehydration at the bridged level after dynamic stabilization [37, 38]. However, severe degeneration of the disc is difficult to reverse. Therefore, patients with a significant reduction in intervertebral disc height were excluded from this study.

There were limitations to this study: lack of a control group, limited patient number, and short follow-up period. Nevertheless, these are common issues when evaluating a new surgical technique. Besides, there was no further analysis of the causes of low back pain before the second surgery and the role of dynamic stabilization in alleviating it. In addition, due to the high costs, only 11 patients received MRI at postoperative follow-up in the study. A longer follow-up period and more patients receiving MRI will contribute to better observation of the disc changes after dynamic stabilization.

## 5. Conclusions

Our results suggest that TDS combined with limited rediscectomy resulted in a safe and effective procedure in middle-aged patients with Carragee type II and type IV RLDH. It was able to stabilize the operated segment with partial motion preservation. Moreover, it could maintain disc height and decrease the risk of recurrence in patients with a large posterior annular defect.

## Figures and Tables

**Table 1:** Demographics of dynamic stabilization for recurrent lumbar disc herniation.

Case no.	Sex	Age (years)	Level	Primary surgery	Recurrence time (months)	Follow-up (months)	Complication
1	F	53	L5-S1	OD	120	28	
2	F	62	L4-5	OD	12	49	
3	M	45	L5-S1	OD	60	54	
4	M	34	L4-5	MED	19	33	Superficial wound infection
5	M	41	L4-5	OD	24	42	
6	M	48	L5-S1	OD	120	30	
7	F	48	L5-S1	MED	9	32	
8	F	40	L4-5	OD	84	30	
9	M	35	L5-S1	MED	36	63	
10	M	58	L4-5	OD	108	40	
11	F	34	L4-5	OD	60	37	
12	M	53	L4-5	OD	96	36	
13	M	52	L4-5	OD	24	46	
14	M	47	L4-5	OD	6	32	
15	F	50	L5-S1	OD	192	56	
16	F	61	L4-5	OD	168	30	
17	M	59	L4-5	OD	60	47	
18	F	58	L4-5	OD	96	38	
19	M	46	L4-5	MED	132	34	Transient low back and hip pain
20	F	52	L5-S1	OD	7	32	
21	M	38	L5-S1	OD	72	33	
22	M	43	L5-S1	OD	24	29	
23	M	54	L5-S1	OD	24	33	
24	M	32	L4-5	OD	30	36	

M: male, F: female, OD: open discectomy, and MED: microendoscopic discectomy.

**Table 2:** Clinical outcomes.

	Preoperative	3 months	Last follow-up	*F*	*P*
VAS_back_	3.8 ± 0.8	1.3 ± 0.6	0.9 ± 0.4	110.49	0.001
VAS_leg_	5.5 ± 1.1	0.9 ± 0.6	0.7 ± 0.5	525.16	0.001
ODI (%)	57.9 ± 10.6	23.2 ± 7.8	12.8 ± 6.2	171.475	0.001

**Table 3:** Radiographic outcomes.

	Preoperative	3 months	Last follow-up	*F*	*P*
Lordosis at instrumented segment (°)	8.0 ± 4.2	6.8 ± 2.4	7.0 ± 2.6	2.379	0.104
Disc height at instrumented segment (mm)	10.4 ± 1.9	9.3 ± 1.9	9.1 ± 1.8	57.562	0.001
ROM at instrumented segment (°)	6.4 ± 3.2	4.0 ± 0.9	4.2 ± 0.6	12.578	0.001
ROM at the 1^st^ proximal segment (°)	9.0 ± 3.9	9.5 ± 2.8	9.9 ± 2.6	1.969	0.151
ROM at L1-S1 (°)	34.1 ± 13.1	29.2 ± 6.8	34.8 ± 10.5	4.496	0.016

## Data Availability

The data used to support the findings of this study are available from the corresponding author upon request.
